# Association of UGT1A6 gene polymorphism with clinical outcome in pediatric epileptic patients on sodium valproate monotherapy

**DOI:** 10.1590/1414-431X2021e11097

**Published:** 2021-06-14

**Authors:** N. Banawalikar, S. Adiga, U. Adiga, V. Shenoy, S. Kumari, P. Shetty, S. Shetty, K.P. Sharmila

**Affiliations:** 1 Central Research Laboratory, KS Hegde Medical Academy, Mangalore, Karnataka, India; 2 Department of Pharmacology, KS Hegde Medical Academy, Mangalore, Karnataka, India; 3 Department of Biochemistry, KS Hegde Medical Academy, Mangalore, Karnataka, India; 4 Department of Pediatrics, KS Hegde Medical Academy, Mangalore, Karnataka, India

**Keywords:** UGT1A6, Genetic polymorphism, Sodium valproate, Clinical outcome

## Abstract

Pediatric epilepsy comprises chronic neurological disorders characterized by recurrent seizures. Sodium valproate is one of the common antiseizure medications used for treatment. Glucuronide conjugation is the major metabolic pathway of sodium valproate, carried out by the enzyme uridine 5′-diphosphate (UDP) glucuronosyl transferase (UGT) whose gene polymorphisms may alter the clinical outcome. The objective of this study was to assess the association between UGT1A6 genetic polymorphism and clinical outcome in terms of efficacy and tolerability in pediatric epileptic patients on sodium valproate monotherapy. Pediatric epileptic patients (n=65) aged 2-18 years receiving sodium valproate monotherapy for the past one month were included. Genetic polymorphism patterns of UGT1A6 (T19G, A541G, A552C) were evaluated by PCR-RFLP. Clinical outcome was seizure control during the 6 months observation period. Tolerability was measured by estimating the hepatic, renal, and other lab parameters. Out of 65 patients, TT (40%), TG (57%), and GG (3%) patterns were observed in UGT1A6 (T19G) gene, AA (51%), AG (40%), and GG (9%) in (A541G) gene, and AA (43%), AC (43%), and CC (14%) in (A552C) gene. No statistical difference in clinical outcome was found for different UGT1A6 genetic polymorphism patterns. We concluded that different patterns of UGT1A6 genetic polymorphism were not associated with the clinical outcome of sodium valproate in terms of efficacy and tolerability. Sodium valproate was well-tolerated among pediatric patients with epilepsy and can be used as an effective antiseizure medication.

## Introduction

Epilepsy is a neurological disorder comprising heterogeneous and complex brain disorders of many seizure types and epilepsy syndromes (1,2). The overall prevalence of epilepsy in India is estimated to be 3-11.9 per 1000 people (3). It is common practice for pediatricians and neurologists to begin long-term, daily antiseizure medications after a patient has experienced unprovoked seizures (4). Anti-epileptic drugs (AEDs) lead to satisfactory seizure control for about 60-70% of newly treated patients (5), while the remaining 30% of patients will have uncontrolled epilepsy with AED treatment for a prolonged period with recurrent seizures, adverse effects, and significantly increased risk of mortality and morbidity (1,5). The goal of epilepsy treatment in the pediatric patient is to prevent the seizure attack for two years with an appropriate antiseizure medication with minimal adverse effects so that the drug can be slowly tapered off later.

Pharmacogenetics refers to the science about the genetic variations affecting drug metabolism, drug targets, or disease pathways leading to a varying response to the drug concerning its efficacy or adverse effects. Hence, it is important to investigate the role of genetic polymorphism that can alter drug metabolism and the effect of antiseizure medications.

Sodium valproate is a widely used broad-spectrum antiseizure medication due to its effect on different types of epilepsy. Glucuronidation and β-oxidation are the prominent metabolic pathways, while CYP-mediated oxidation is a minor pathway in the metabolization of sodium valproate drugs. Glucuronidation is catalyzed by uridine-5'diphosphate glucuronosyl transferases (UGTs), and polymorphisms affecting genes encoding for UGT enzymes may result in alterations of sodium valproate metabolism. Changes in the glucuronidation rate of sodium valproate may alter the blood level of the drug resulting in either insufficient plasma concentration that leads to reduced effectiveness of therapy or elevated plasma concentration of the drug that manifests as toxicity (6). Previous research has focused on UGT1A6 and CYP polymorphisms in the Chinese, Iranian, and Indian populations. Most of the studies have aimed at finding the pattern of genetic polymorphism and its effect on the serum concentration of the patient. However, currently available information on sodium valproate on UGT1A6 polymorphism and its association with clinical outcome in the Indian population is very limited.

The objectives of this study were to evaluate the pattern of UGT1A6 gene polymorphism in pediatric epileptic patients and assess the association of UGT1A6 gene polymorphism with clinical outcome in terms of efficacy and tolerability in pediatric epileptic patients on sodium valproate monotherapy.

## Material and Methods

This cohort study was carried out at the pediatric department of Justice KS Hegde Charitable Hospital and Central Research Laboratory of KS Hegde Medical Academy, Mangalore, India, from February 2018 to December 2019. Sixty-five pediatric epileptic patients aged 2-18 years of either sex treated with sodium valproate monotherapy were enrolled. The Ethics Committee approval was obtained before the study. Written informed consent was obtained before enrollment. Inclusion criteria were patients diagnosed clinically by EEG and who were on a steady stable dose of 20 mg/kg per day sodium valproate twice/thrice a day for the past one month. Patients with a history or evidence of hepatitis or impaired renal functions or who were already on treatment with any other antiseizure medications or drugs that induced or inhibited sodium valproate metabolism were excluded.

Four milliliters of whole blood was collected with aseptic precautions once the patient fulfilled the criteria and during a 6-month follow-up visit. The sample was separated into 2 parts: 2 mL of EDTA whole blood was stored at -80°C for genetic polymorphism analysis and 2 mL of whole blood in a plain vial was centrifuged at 1968 *g* for 5 min at 25°C to obtain serum, and then stored at -80°C to analyze biochemical parameters. Liver function tests (albumin, total protein, total and direct bilirubin, serum glutamic oxaloacetic transaminase (SGOT), serum glutamic pyruvic transaminase (SGPT), alkaline phosphatase (ALP)), renal function test (blood urea, serum creatinine), platelet count, and serum amylase were estimated at the time of enrollment and at 6 months.

### Genotyping

**Figure 1:**
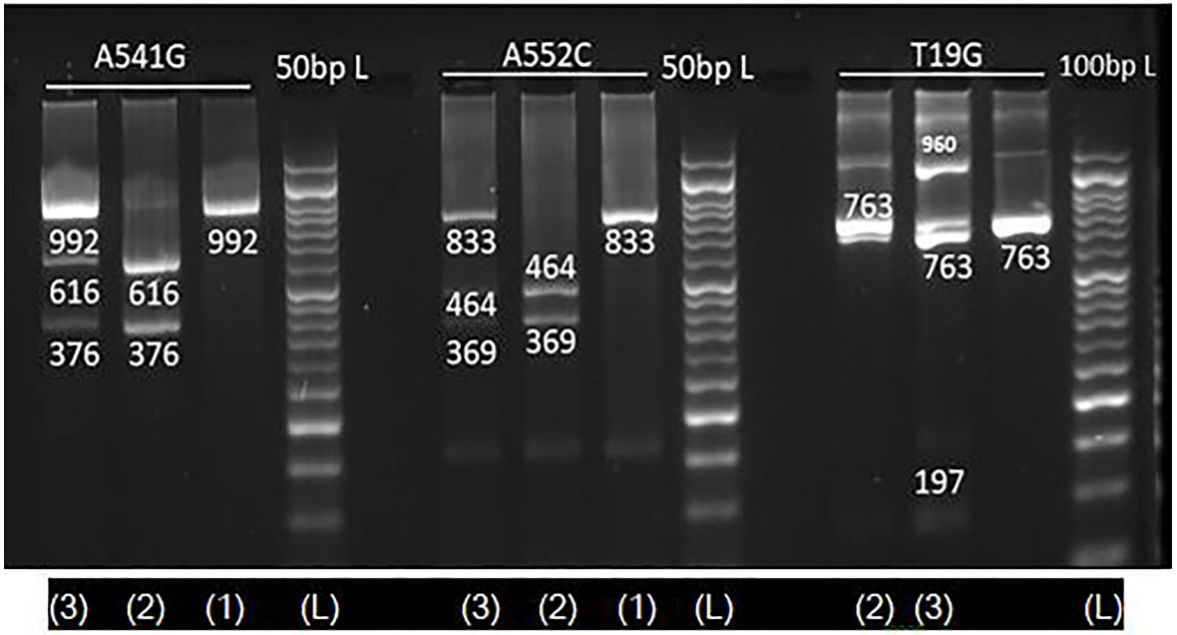
Electrophoresis gel image patterns of UGT1A6 (A541G, A552C, T19G) gene after digestion with *NsiI*, *Fnu4*HI, and *Hha*I, respectively. L: Ladder; lane (1): homozygosity for variant allele; lane (2): homozygosity for common allele; lane (3): heterozygosity.

Genomic DNA from whole blood was isolated by phenol-chloroform extraction and ethanol precipitation method. The UGT1A6 (T19G, A541G, A552C) polymorphisms were analyzed by the polymerase chain reaction-restriction fragment length polymorphism (PCR-RFLP) method (7,8). Amplification was performed in an MJ-Mini Thermal cycler (BioRad, Japan). PCR was conducted with an initial denaturation enzyme activation step at 95°C for 5 min, amplification step for 35 cycles at 95°C for 30 s, an annealing temperature for 30 s, treatment at 72°C for 30 s, and a final extension step at 72°C for 5 min. PCR product was digested with each restriction enzyme (*Hha* I, NsiI, *Fnu4*HI), respectively, for T19G, A541G, A552C (Figure 1). The amplified product of DNA samples and restriction fragments was separated on a 2% agarose gel with ethidium bromide. The genotype was assigned based on the results of the analysis of the digestion patterns.

Parents were instructed to maintain a diary to record seizure attacks if any, duration of the attack, and change in the pattern from previous episodes, and the days the child had skipped the medication. They were also instructed to bring the child for a clinical visit in the event of nausea, vomiting, loss of appetite, jaundice, abdominal pain anytime or otherwise at the end of 6 months.

Clinical outcome was assessed in terms of efficacy and safety of sodium valproate. The drug efficacy was measured in terms of treatment responders or non-responders. Responder to sodium valproate was defined as patients with no relapse or 1 episode of seizure attack in 6 consecutive months of follow-up and who tolerated sodium valproate monotherapy well without any adverse effect. Non-responders were those with two or more episodes of seizure attack in six months of follow-up or with any drug adverse effect after therapy initiation (9). Tolerability was assessed by comparing the biochemical parameters between baseline and at the six-month visit and by assessing whether patients experienced jaundice, pancreatitis, or signs of thrombocytopenia.

### Sample size calculation

Based on the genotype frequencies reported previously, considering a standard drug concentration of 23 µg/mL (10 [Bibr B11]), and fixing the margin of error as 5, the calculated sample size was 87.

However, due to the change in treatment guidelines of pediatric epilepsy given by the Indian Pediatric Association (preference of levetiracetam over sodium valproate), we could enroll only 65 patients.

### Statistical analysis

The collected data are reported as frequency and percentage for qualitative data and as means±SD for quantitative data using SPSS software (version 16.0, IBM, USA). Comparison between responders and non-responders in different gene patterns of UGT1A6 studied was done by the chi-squared test. Biochemical parameters were compared between baseline and at 6 months follow-up by paired *t*-test in different age groups. A P-value <0.05 was considered statistically significant.

## Results

The mean age of patients enrolled in the study was 7.38±3.67 years. There were 34 male and 31 female patients and the mean body mass index was 16.89±4.56 kg/m^2^.

Out of 65 patients, TT (40%), TG (57%), and GG (3%) patterns were observed in UGT1A6 (T19G) gene, AA (51%), AG (40%), and GG (9%) in (A541G) gene, and AA (43%), AC (43%), and CC (14%) in (A552C) gene. The frequencies were consistent with the Hardy-Weinberg equilibrium (Table 1). No statistical difference was found in clinical outcome with UGT1A6 (T19G, A541G, A552C) genetic polymorphisms (Table 2). There was a higher pattern of mutant alleles carriers for the genes UGT1A6 (T19G and A552C).

**Table 1:** Hardy-Weinberg equilibrium test for UGT1A6 gene.

Genotype	Actual values	Expected values	P-value
T19G			
TT	26	30.5	6.58
TG	37	28.0	
GG	2	6.5	
A541G			
AA	33	32.6	0.0716
AG	26	26.9	
GG	6	5.5	
A552C			
AA	28	27.1	0.2184
AC	28	29.7	
CC	9	8.2	

The chi-squared test was used for statistical analysis.

**Table 2:** Association of UGT1A6 gene polymorphisms with clinical outcome (N=65).

Genotype UGT1A6	Polymorphism pattern	Polymorphism	Clinical outcome		P-value
			Responders (N=53) (81.5%)	Non-responders (N=12) (18.5%)	
T19G	TT	40%	20 (37.8%)	6 (50%)	0.318
	TG	57%	32 (60.4%)	5 (41.6%)	
	GG	3%	1 (1.8%)	1 (8.4%)	
A541G	AA	51%	28 (52.8%)	5 (41.6%)	0.568
	AG	40%	21 (39.6%)	5 (41.6%)	
	GG	9%	4 (7.6%)	2 (16.8%)	
A552C	AA	43%	23 (43.4%)	5 (41.6%)	0.952
	AC	43%	23 (43.4%)	5 (41.6%)	
	CC	14%	7 (13.2%)	2 (16.8%)	

Data are reported as n (%). Chi-squared test.

A total of 53 patients (81.5%) responded well to the treatment without any relapse of seizure and 12 patients (18.5%) had a relapse of seizure with two or more episodes. These patients were shifted to other antiseizure medications. No statistical difference was found in clinical efficacy (seizure control) with UGT1A6 (T19G, A541G, A552C) genetic polymorphism (Table 2). We have also analyzed the relative risk between the clinical outcome among carriers of wild and mutant allele types in UGT1A6 (T19G, A541G, and A552C). The wild-type gene pattern patients in A541G seemed to have a better response in seizure control compared to mutant allele type carriers even though the P-value was not statistically significant. It was also observed that the wild-type pattern seemed to have a poor response in terms of efficacy at T19G and A552C loci (Table 3).

**Table 3:** Relative risk of a poor clinical outcome in various gene polymorphisms.

Gene loci	Pattern	Probability of poor response	Clinical outcome (N, %)		Relative risk	P-value
			Good (N=53) (81.5%)	Poor (N=12) (18.5%)		
T19G	Wild	0.23	20 (37.8%)	6 (50%)	1.53	0.52
	Mutant	0.15	33 (62.2%)	6 (50%)		
A541G	Wild	0.15	28 (52.8%)	5 (41.6%)	0.71	0.54
	Mutant	0.21	25 (47.2%)	7 (58.4%)		
A552C	Wild	0.17	23 (43.4%)	5 (41.6%)	1.01	>0.99
	Mutant	0.18	30 (56.4%)	7 (58.4%)		

Data are reported as n (%). Fisher's exact test.

Fifty-two patients (80%) were diagnosed with generalized tonic-clonic seizures (GTCS) and 13 patients (20%) were diagnosed with focal seizures with awareness impairment (FSAI). There was no statistically significant difference in relative risk of poor clinical outcome among patients of GTCS and FSAI of different gene patterns (Table 4). However, GTCS patients with T19G and A552C of wild-type allele had a higher risk of having a poor response (poor seizure control) compared to FSAI patients.

**Table 4:** Relative risk of poor clinical outcome in various types of seizures.

Gene loci	Seizure type	Gene pattern	Clinical outcome		Relative risk	P-value
			Good N=52 (80%)	Poor N=13(20%)		
T19G	GTCS	Wild	11 (21.2%)	5 (38.5%)	2.25	0.25
		Mutant	30 (57.7%)	6 (46.1%)		
	FSAI	Wild	9 (17.3%)	1 (7.7%)	0.42	0.30
		Mutant	2 (3.8%)	1 (7.7%)		
A541G	GTCS	Wild	22 (42.4%)	5 (38.5%)	0.92	>0.99
		Mutant	19 (36.5%)	6 (46.1%)		
	FSAI	Wild	6 (11.5%)	0	0	0.46
		Mutant	5 (9.6%)	2 (15.4%)		
A552C	GTCS	Wild	16 (30.8%)	5 (38.5%)	1.47	0.50
		Mutant	25 (48.1%)	6 (46.1%)		
	FSAI	Wild	7 (13.5%)	0	0	0.19
		Mutant	4 (7.6%)	2 (15.4%)		

Data are reported as n (%). Fisher's exact test. GTCS: generalized tonic-clonic seizures; FSAI: focal seizures with awareness impairment.

Except for two patients, all children had normal liver, hematological, and renal function parameters at baseline and 6 months follow-up (Table 5). One patient developed acute pancreatitis, with elevated serum lipase and features suggestive of pancreatitis in ultrasound. De-challenge was given and the patient recovered completely and shifted to levetiracetam. Causality assessment was done using the Naranjo algorithm, which revealed a score of +6 suggesting the probable nature of the adverse drug effect was from sodium valproate.

**Table 5:** Biochemical parameters between baseline and 6 months in the different age groups.

Parameters	2 years			6-12 years			12-18 years		
	Basal	6 months	P-value	Basal	6 months	P-value	Basal	6 months	P-value
Albumin (g/dL)	4.3±0.33	4.2±0.33	0.674	4.5±0.39	4.4±0.26	0.363	4.6±0.28	4.6±0.24	0.984
Total protein (g/dL)	6.8±0.37	7.2±0.60	0.117	7.4±0.74	7.4±0.5	0.974	7.5±0.49	7.7±0.3	0.745
SGOT (IU/L)	33.8±10	28.3±7.8	0.215	27.2±7	26.9±9.5	0.04*	26.7±15	23.2±5	0.326
SGPT (IU/L)	14.7±6	14.5±7.2	0.091	11.6±4	12±4.8	0.009*	16.3±16	13.1±3.6	0.173
ALP (IU/L)	219±93	186±49	0.881	198±53	217±76	0.267	177±84	158±78	0.578
Bil Dir (mg/dL)	0.07±0.03	0.09±0.04	0.565	0.16±0.1	0.11±0.05	0.034*	0.14 ±0.13	0.12±0.08	0.028*
Bil Tot (mg/dL)	0.2±0.1	0.2±0.1	0.496	0.34±0.3	0.32±0.14	0.002*	0.38±0.2	0.32±0.2	0.032*
Urea (mg/dL)	23.6±9.4	19.2±9.1	0.928	21.3±6.5	21.3±7.4	0.850	20.1±7.7	20.1±7.2	0.915
Creat (mg/dL)	0.40±0.2	0.35±0.2	0.242	0.53±0.2	0.36±0.1	0.017*	0.64±0.2	0.53±0.1	0.119
Amylase (IU/L)	84.4±32	73.3±29	0.301	93.3±54	84.8±27	0.012*	87.3±36	90.3±44	0.038*
Platelet (Lk/cum)	2.9±0.33	2.7±0.47	0.167	2.8±0.78	2.5±0.45	0.342	2.8±0.32	2.7±0.35	0.092

Data are reported as means±SD. SGOT: serum glutamic oxaloacetic transaminase; SGPT: serum glutamic pyruvic transaminase; ALP: alkaline phosphatase; Bil Dir: direct bilirubin; Bil Tot: total bilirubin. *P≤0.05 (paired *t*-test). Bold type indicates statistical significance.

### Safety parameters

We divided the biochemical and hematological parameters into three subgroups as there may be variations by age group. A statistically significant difference was found in the values at 6-month assessment compared to baseline values in SGOT, SGPT, serum amylase, direct bilirubin, total bilirubin, and serum creatinine in the age group of 6-12 years. In the age group of >12-18 years, direct bilirubin, total bilirubin, and serum amylase values were statistically different between baseline and 6 months. Except for one patient who developed acute pancreatitis, all the remaining patients had no adverse effect. Three patients had elevated SGOT and two had elevated ALP levels, however, only one patient had severe abdominal pain, later found to have acute pancreatitis.

## Discussion

Many researchers have investigated the role of gene polymorphisms in the pharmacokinetics and pharmacodynamics aspects of sodium valproate. The pattern of UGT1A6 gene polymorphism was slightly different in the Indian population compared to the Chinese population concerning UGT1A6 (T19G, A541G, A552C type) (Table 6) (10–17). In the Chinese pediatric epileptic population, the predominant genetic polymorphism pattern observed was wild type while in our study population the predominant pattern observed was mutant type. However, there was no difference in the genetic pattern between our patients (South Indian) and North Indian pediatric epileptic patients (8,16).

**Table 6:** Prevalence of UGT1A6 gene polymorphisms in children with epilepsy of various populations based on published studies.

Genotype UGT1A6	Chinese				Japanese	Caucasian	Indian	Indian
	Hung et al. (10) (n=162)	Guo et al. (11) (n=98)	Chu et al. (12) (n=242)	Xing et al. (13) (n=534)	Saeki et al. (14) (n=195)	Lampe et al. (15) (n=100)	Jain et al. (16) (n=80)	Current study (n=65)
T19G								
TT	62.4%	54.3%	NS	NS	NS	NS	45%	40%
TG	30.9%	39.4%	NS	NS	NS	NS	39%	57%
GG	6.8%	6.4%	NS	NS	NS	NS	16%	3%
A541G								
AA	64.8%	56.4%	65%	60%	62%	46%	49%	51%
AG	29.6%	39.4%	33%	35%	33%	43%	39%	40%
GG	5.6%	4.3%	2%	5%	5%	11%	12%	9%
A552C								
AA	62.9%	55.3%	57%	57%	NS	48%	44%	43%
AC	30.3%	39.4%	39%	37%	NS	42%	40%	43%
CC	6.8%	4.3%	4%	6%	NS	10%	16%	14%

NS: not studied.

Different studies have reported different observations as to UGT gene polymorphism and its effect on sodium valproate concentration. Jain et al. (16), Wang et al. (18), and Chu et al. (12 [Bibr B13] [Bibr B14] [Bibr B15]) reported no significant effect of polymorphisms on serum valproate concentrations. None of the available literature to date has explained the influence of UGT genetic variants on the clinical outcome of sodium valproate monotherapy. However, Hung et al. (10) reported that patients showing good drug response (seizure-free/good seizure control) should be evaluated for multiple genetic influences including UGT1A6 gene polymorphism on sodium valproate to guide dosing, seizure control, and adverse drug reactions. The authors concluded that carriers of the variant UGT1A6 19T>G, 541A>G, and 552A>C alleles tend to require higher sodium valproate dosages and lower concentration-to-dose ratios (CDRs) than non-carriers (P<0.0001) and the homozygous carriers also seemed to require higher sodium valproate dosages and lower CDRs (P<0.0001).

Munisamy et al. (7) reported that UGT1A6 552A>C polymorphism plays a significant role in the elimination half-life, which was longer, and the clearance rate was lower in poor metabolizers at a steady-state concentration of sodium valproate. This showed toxicity in the intermediate metabolizers group or the extensive metabolizers group and it, thereby, has an impact on the toxicity of the sodium valproate drug found in the North Indian epileptic population. Jain et al. (16) reported that there was no significant association between sodium valproate doses, serum valproate concentration, and UGT1A6 genotypes in the North Indian population of children with epilepsy. Algharably et al. (19) reported in an Egyptian study that gene polymorphism of UGT1A6 at 541A>G and 552A>C loci may influence the clinical response profile of pediatric epileptic patients including both adverse drug reactions and seizure activity. According to our study, the non-responders of A541G wild type was 15.16% and the mutant, it was 21.82% at the end of 6 months. A study conducted in the Egyptian pediatric population showed 47% non-response in wild-type and 52.9% in mutant-type carriers. The non-responder rate was 17.79% in patients with wild-type patterns in the A552C loci and 18.92% in patients with mutant type. A study done in the Egyptian pediatric population showed 55.9% non-response in wild-type and 44.1% in mutant-type carriers. However, in our study, we could not find any association between the different patterns of polymorphism with clinical efficacy and tolerability.

The limitation of our study was the small sample size. In the present context of the changed protocol in the treatment of pediatric epilepsy (preference of levetiracetam over sodium valproate) only metacentric studies involving different regions may answer our limitation and add quality to the data in terms of genetic pattern.

We conclude that different patterns of UGT1A6 genetic polymorphism were not associated with the clinical outcome of sodium valproate in terms of efficacy and tolerability in epileptic pediatric patients. Sodium valproate was well tolerated and can be used as an effective antiseizure medication in this population.
